# COVID-19 pandemic: Regarding alcohol consumption

**DOI:** 10.1192/j.eurpsy.2021.784

**Published:** 2021-08-13

**Authors:** M.J. Gonçalves, L. Linhares, C. Sereijo, R. Saraiva, F. Ismail

**Affiliations:** 1 Psychiatry, Centro Hospital Lisboa Norte, Lisboa, Portugal; 2 Psiquiatria, Centro Hospitalar Universitario Lisboa Norte, Lisboa, Portugal; 3 Psiquiatria, Centro Hospitalar Universitario Lisboa Norte, oeiras, Portugal

**Keywords:** COVID-19, alcohol use disorders (AUD), alcohol consumption

## Abstract

**Introduction:**

On March, 2020, the World Health Organization (WHO) declared the COVID-19 outbreak a global pandemic. Social isolation, unemployment and financial difficulties can have an impact on mental health and trigger the use of alcohol as a form of coping. Since the beginning of this pandemic, the WHO had warned the general public of the potential risks of increased alcohol consumption, which might result in a higher incidence of alcohol use disorders (AUD) in future.

**Objectives:**

The aim is to do a review of the literature of alcohol consumption during the COVID-19 pandemic.

**Methods:**

Non-systematic review of the literature with selection of scientific articles published in the last 7 months; by searching the Pubmed databases, the following MeSH terms were used: COVID-19; alcohol consumption.

**Results:**

A recent article in The Lancet suggested that mental health and alcohol use during the pandemic, a major public health concern, are worthy of attention. Market research showed that alcohol sales increased in several countries compared to the same time last year. However, with the closure of several drinking places, sales are not in themselves reliable enough estimates of alcohol consumption. On the other hand, economic crises can lead to a reduction in alcohol consumption, due to financial problems or the risk of unemployment.

**Conclusions:**

The present global circumstance is unique, and there is a need for further research on the relationship between alcohol consumption and COVID-19 to understand its long-term effects and develop specific prevention programs for this population.

